# Short-term change of carotid intima-media thickness after treatment of hyperglycemia in patients with diabetes: a cross-sectional study

**DOI:** 10.1186/s13104-016-2080-9

**Published:** 2016-05-23

**Authors:** Ayumi Tenjin, Yoshio Nagai, Sayaka Yuji, Satoshi Ishii, Hiroyuki Kato, Akio Ohta, Yasushi Tanaka

**Affiliations:** Division of Metabolism and Endocrinology, Department of Internal Medicine, St. Marianna University School of Medicine, 2-16-1, Sugao, Miyamae-ku, Kawasaki, Kanagawa 216-8511 Japan

**Keywords:** Carotid artery intima-media thickness, Hemodynamics, Diabetes mellitus

## Abstract

**Background:**

The carotid artery intima-media thickness (CIMT) has been used as a predictor of cardiovascular events, but it remains unclear whether CIMT can change over the short term. We evaluated changes of CIMT in patients with diabetes during admission to hospital for 2 weeks.

**Methods:**

A total of 279 inpatients with diabetes aged 61 ± 14 years were recruited. They were on treatment with insulin and/or oral agents, excluding drugs that influence the fluid balance and hemodynamics. CIMT was measured on the day after admission and on the day before discharge, and the association of ΔCIMT (calculated by subtracting the baseline value from that on the day before discharge) with clinical factors was evaluated.

**Results:**

Based on the reported annual increase of CIMT (0.04 mm/year), the patients were divided into three groups, in which CIMT increased [I: ΔCIMT ≥ 0.04 mm, n = 64, ΔCIMT = 0.077 ± 0.048 (mean ± SD)], CIMT decreased (D: ΔCIMT ≤ −0.04 mm, n = 51, ΔCIMT = −0.090 ± 0.086), or CIMT was unchanged (N: −0.04 mm < ΔCIMT < 0.04 mm, n = 164, ΔCIMT = 0.002 ± 0.022). Binary logistic regression analysis showed that baseline CIMT and hemoglobin (Hb) were positively correlated, while Hb on the day before discharge was negatively correlated, with a decrease of CIMT. In contrast, baseline HbA1c and Hb were negatively correlated, while Hb on the day before discharge was positively correlated, with an increase of CIMT.

**Conclusions:**

CIMT may show plasticity in patients with diabetes and can change even after short-term treatment of hyperglycemia for 2 weeks.

## Background

The carotid artery intima-media thickness (CIMT) is an ultrasound biomarker of subclinical atherosclerosis that predicts events related to cardiovascular disease (CVD). The Kuppio ischemic heart disease study first reported an association of CIMT with future coronary artery events [1]. Subsequently, various large-scale prospective studies have confirmed the usefulness of CIMT for assessing the risk of CVD events [2–4]. CIMT has been reported to be larger in diabetic patients than in non-diabetic subjects [5]. Previous studies have also shown that the annual rate of CIMT increase in Japanese patients with type 2 diabetes is about 0.04 mm [6–8]. Previous intervention studies have demonstrated a decrease of CIMT or attenuation of its progression in patients with diabetes after treatment of hyperglycemia [7, 8]. These changes of CIMT were observed after at least 6 months, but lipid-lowering intervention studies using statins for 6–8 weeks have found a decrease of CIMT [9, 10], suggesting that more rapid change may be possible.

CIMT measures both the intimal and medial layers of the carotid artery, with the former comprising the endothelial cell layer plus the subendothelial space and the latter consisting of smooth muscle cells with interstitial fibrous tissue. Recent studies have shown that the mechanism of medial thickening may involve remodeling of smooth muscle cells by hyperplasia and/or hypertrophy due to hemodynamic changes caused by increased stroke volume or glucose-related arterial stiffness [11]. Severe hyperglycemia is generally accompanied by dehydration due to persistent osmotic diuresis. Thus, hemodynamics may change rapidly during treatment of either severe or mild hyperglycemia in patients with diabetes. Accordingly, we hypothesized that CIMT may show short-term alterations in response to such hemodynamic changes. To investigate this hypothesis, we evaluated short-term changes of CIMT in patients with diabetes from the day after admission for treatment of hyperglycemia to the day before discharge (about 14 days later).

## Methods

### Subjects

This was a cross-sectional study carried out at St. Marianna University Hospital (Kawasaki, Japan). A total of 312 consecutive patients admitted for treatment of type 1 or type 2 diabetes between April 2007 and January 2015 were recruited. All of the patients were admitted for initial diabetes education and improvement of glycemic control under a program provided by the Japanese health system, and none of them were hospitalized for acute metabolic complications (e.g. diabetic ketoacidosis). Patients with malignancy, severe heart failure (NYHA classes II–IV), severe acute or chronic kidney disease (CKD stages 4 and 5), liver cirrhosis or active hepatitis, a history of steroid therapy, endocrine disease, and pregnancy or breast-feeding were excluded, as were any other patients judged to be unsuitable for this study. Patients were treated with insulin and/or oral hypoglycemic agents, but the following medications were not used to avoid an influence on the fluid balance and hemodynamics: thiazolidinediones, sodium glucose co-transporter 2 inhibitors, angiotensin-converting enzyme inhibitors, angiotensin-II receptor blockers, and diuretics.

### Ethics approval and consent to participate

The study protocol was approved by the ethics committee of St. Marianna University, and all patients provided written informed consent to participate.

### Data collection

In all patients, the height, body weight (BW), blood pressure (BP), and CIMT were determined at baseline. Blood samples were collected in the morning on the day after admission following an overnight fast (at least 12 h). Fasting plasma glucose (FPG) was measured by the hexokinase UV method, hemoglobin A1c (HbA1c) was determined by the latex aggregation method, and serum glycated albumin (GA) was assayed by an enzymatic method using an albumin-specific protease (ketoamine oxidase) and an albumin assay reagent (Lucica GA-L, Asahi Kasei Pharma, Tokyo, Japan). Plasma lipids (total cholesterol, triglycerides, HDL-cholesterol, and LDL-cholesterol) were determined by enzymatic methods. The daily glucose profile was evaluated by 10-point self-monitoring of blood glucose (SMBG) using a onetouch ultra blood glucose monitor (Life Scan, Milpitas, CA, USA). On the morning of the day before discharge (about 10–14 days after admission), the BW, BP, and CIMT were measured, blood samples were obtained, and 10-point SMBG was repeated under the same conditions.

### Measurement of CIMT

Assessment of CIMT was performed by a single trained sonographer (SY, a Registered Medical Sonographer certified by the Japan Society of Ultrasonics in Medicine) using the same ultrasonography device (LOGIQe, GE Yokokawa Medical, Tokyo, Japan) and a 10 MHz linear transducer with high axial resolution in the order of 0.01 mm. Patients were examined in the supine position. B-mode scanning of the common carotid artery (CCA) was performed bilaterally in two different longitudinal projections (anterior-oblique and lateral), and 20 mm long images of the far wall of the CCA were obtained from the origin of the carotid bulb to a point 20 mm proximal. Then CIMT was automatically determined from these images by using analytical software (Intimascope 5.01R, Soft Medical Co. Ltd., Tokyo, Japan) [12–14], which was designed to divide a 2 cm length of the CCA into 330 points for CIMT measurement and calculate the average of the 330 values thus obtained. We averaged the four CIMT values thus obtained (from the two projections on the right and left sides) to calculate the mean CIMT for use in subsequent analyses. The change of the mean CIMT (ΔCIMT) was calculated by subtracting the baseline mean CIMT from that determined on the day before discharge. Thus, a positive value means CIMT gets larger (worsens) and a negative value means CIMT gets smaller (improves). It was previously reported that the annual rate of CIMT increase in Japanese patients with type 2 diabetes is about 0.04 mm [6–8]. Based on this finding, we classified the patients into three groups, in which CIMT increased (group I, ΔCIMT ≥ 0.04 mm), CIMT decreased (group D, ΔCIMT ≤ −0.04 mm), or CIMT was unchanged (group N, −0.04 mm < ΔCIMT < 0.04 mm).

### Statistical analysis

Results are expressed as the mean ± SD. Comparison of mean values among the three groups was done by univariate ANOVA followed by a post hoc Tukey multiple comparison test. To assess the relationship of clinical factors with an increase or decrease of CIMT, binary logistic regression analysis was performed. All analyses were done with SPSS ver. 21.0 software (IBM, Tokyo, Japan), and statistical significance was defined as P < 0.05.

## Results

A total of 279 patients completed the study, while 33 patients dropped out because they were discharged from hospital earlier than 10 days. Baseline clinical characteristics of the subjects are displayed in Table 1. The three groups showed no differences of gender, age, and body mass index (BMI). In group D, CIMT and FPG were both higher than in group N or group I. HbA1c and GA were also higher in group D than in group I, while GA was lower in group I than in group N. The other baseline clinical factors did not differ among the three groups.

**Table 1:** Baseline clinical characteristics of the patients

Group	Total	I	N	D	p value
n (%)	279 (100)	64 (22.9)	164 (58.8)	51 (18.3)	
Women (%)	117 (42)	29 (45)	67 (41)	21 (41)	0.823
Age (years)	60.6 ± 14.3	61.6 ± 13.5	59.6 ± 15.2	62.6 ± 11.7	0.333
BMI (kg/m^2^)	25.9 ± 5.2	27.3 ± 5.4	25.3 ± 5.2	26.0 ± 4.2	0.238
CIMT (mm)	0.737 ± 0.154	0.726 ± 0.146	0.715 ± 0.148	0.822 ± 0.154*,**	<0.001
Hb (g/dl)	13.5 ± 1.9	13.5 ± 1.8	13.5 ± 2.0	13.6 ± 1.5	0.934
Ht (%)	40.6 ± 5.5	40.7 ± 5.2	40.5 ± 6.0	41.0 ± 4.3	0.813
Fasting plasma glucose (mg/dl)	171.8 ± 53.4	168.5 ± 50.8	166.2 ± 51.8	193.6 ± 56.8*,***	0.005
Mean daily blood glucose (mg/dl)	217.6 ± 61.8	212.7 ± 57.9	214.4 ± 60.1	234.4 ± 69.6	0.092
AUC daily blood glucose	2785 ± 821	2756 ± 805	2731 ± 765	3010 ± 997	0.171
Hemoglobin A1c (%) (mmol/mol)	9.3 (78.6) ± 2.3 (25.6)	8.6 (70.9) ± 1.8 (20.1)	9.4 (79.6) ± 2.5 (26.9)	10.0 (85.4) ± 2.3 (25.2)**	0.007
Glycoalbumin (%)	26.0 ± 8.4	22.9 ± 5.9*	26.6 ± 9.0	27.8 ± 8.3**	0.003
Systolic blood pressure (mmHg)	126.8 ± 19.3	132.6 ± 24.0	124.4 ± 17.9	125.9 ± 14.8	0.107
Diastolic blood pressure (mmHg)	77.6 ± 13.1	79.4 ± 13.1	77.4 ± 12.9	75.6 ± 14.0	0.515
Total cholesterol (mg/dl)	190.9 ± 41.5	188.1 ± 40.1	191.8 ± 41.6	191.6 ± 43.6	0.829
LDL cholesterol (mg/dl)	115.9 ± 35.0	118.2 ± 36.2	114.6 ± 34.2	117.0 ± 36.4	0.767
HDL cholesterol (mg/dl)	47.2 ± 14.5	45.0 ± 10.6	47.8 ± 16.0	48.3 ± 13.5	0.364
Triglycerides (mg/dl)	156.0 ± 116.5	144.9 ± 67.8	160.2 ± 126.7	156.2 ± 129.7	0.683

Values are presented as the mean ± SD or number (%)

*I* increased IMT group, *N* unchanged IMT group, *D* decreased IMT group, *Ht* hematocrit

* p < 0.01 vs N, ** p < 0.01 vs I, *** p < 0.05 vs I

**Fig. 1:**
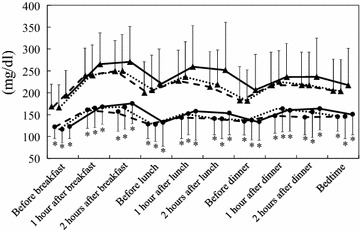
Daily glucose profiles at baseline and on the day before discharge among the three groups. Values are presented as the mean ± SD. *p < 0.01 vs. baseline.  I at admission,  D at admission,  N on the day before discharge,  N at admission,  I on the day before discharge,  D on the day before discharge

**Fig. 2:**
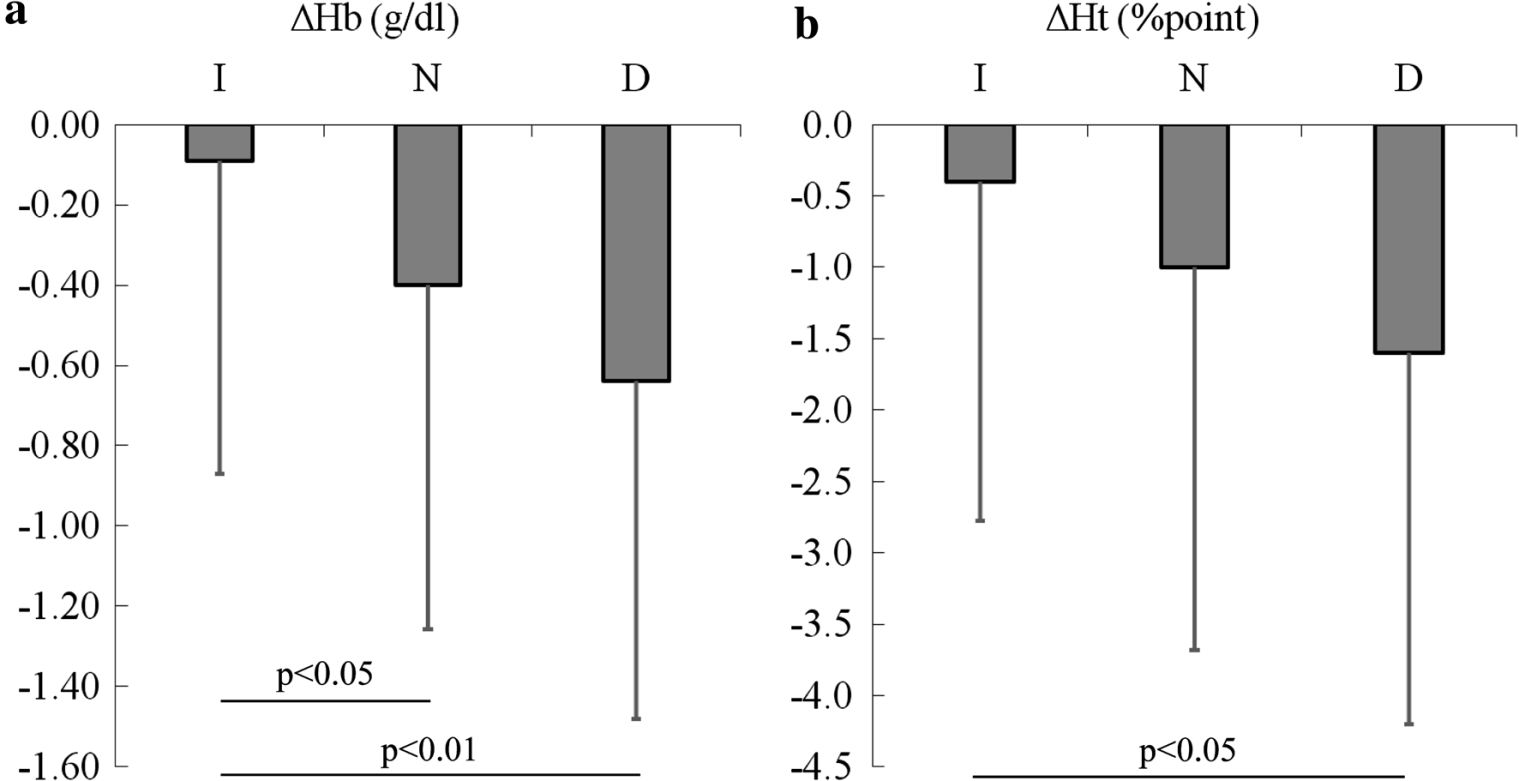
Comparison of the changes of Hb and hematocrit (Ht) among the three groups: **a** ΔHb, **b** ΔHt. Values are presented as the mean ± SD. ΔHb and ΔHt were obtained by subtracting the baseline value from that on the day before discharge

Changes of the daily glucose profile from baseline to the day before discharge are displayed in Fig. 1. Mean SMBG values were lower at all points on the day before discharge in each group, and no differences were observed at the same time points among the three groups. Comparison of clinical factors between baseline and the day before discharge is shown in Table 2. In group I, CIMT was larger than in group N on the day before discharge. ΔCIMT was larger in group I than in group N, while it showed a negative value (decrease of CIMT) in group D (0.077 ± 0.049, 0.002 ± 0.022, and −0.090 ± 0.086 mm, respectively, p < 0.001). The Hb, hematocrit (Ht), and area under the daily glucose profile curve (AUCglu) were not different among the three groups either at baseline or on the day before discharge. BP also did not differ among the three groups at both times. However, ΔHb and ΔHt (difference from baseline to the day before discharge) were larger in group D than in group I, and ΔHb was also larger in group N than in group I (Fig. 2). Since baseline glycemic control markers (such as HbA1c or GA) and ΔHb or ΔHt may be associated with short-term changes of CIMT, binary logistic regression analysis was performed to assess the relationship of baseline CIMT, baseline HbA1c, and Hb at baseline or on the day before discharge with the decrease of CIMT (ΔCIMT ≤ −0.04 mm or not) or increase of CIMT (ΔCIMT ≥ 0.04 mm or not), and the results are shown in Table 3. Baseline CIMT and Hb were positively correlated with a decrease of CIMT, while Hb on the day before discharge was negatively correlated with a decrease of CIMT. Conversely, baseline HbA1c and Hb were negatively correlated with an increase of CIMT, while Hb on the day before discharge was positively correlated with an increase of CIMT.

**Table 2:** Clinical characteristics at baseline and discharge

Group	Total	I	N	D	p value
CIMT at baseline (mm)	0.737 ± 0.154	0.724 ± 0.146	0.714 ± 0.149	0.821 ± 0.154*,**	<0.001
CIMT on the day before discharge (mm)	0.738 ± 0.155	0.800 ± 0.166*	0.715 ± 0.148	0.731 ± 0.145	<0.001
Hb at baseline (g/dl)	13.5 ± 1.9	13.5 ± 1.8	13.5 ± 2.0	13.6 ± 1.5	0.934
Hb on the day before discharge (g/dl)	13.2 ± 1.7	13.4 ± 1.7	13.1 ± 1.8	13.0 ± 1.3	0.305
Ht at baseline (%)	40.6 ± 5.5	40.7 ± 5.2	40.5 ± 6.0	41.0 ± 4.3	0.813
Ht on the day before discharge (%)	39.6 ± 5.0	40.3 ± 4.9	39.5 ± 5.4	39.4 ± 3.7	0.447
AUC daily blood glucose at baseline	2785 ± 821	2756 ± 805	2731 ± 765	3010 ± 997	0.171
AUC daily blood glucose on the day before discharge	1892 ± 398	1846 ± 327	1899 ± 400	1936 ± 486	0.628

Values are presented as the mean ± SD or number (%)

*I* increased IMT group, *N* unchanged IMT group, *D* decreased IMT group, *Ht* hematocrit

* p < 0.01 vs N, ** p < 0.01 vs I

**Table 3:** Logistic regression analysis of factors affecting ΔCIMT

	CIMT decrease (Δ CIMT ≤ −0.04 mm)			CIMT increase (Δ CIMT ≥ 0.04 mm)		
	B	β	p value	B	β	p value
CIMT at baseline (mm)	4.458	0.686	0.000	−0.217	−0.033	0.827
Hb at baseline (g/dl)	0.496	0.936	0.016	−0.388	−0.732	0.041
Hb on the day before discharge (g/dl)	−0.527	−0.895	0.018	0.542	0.92	0.007
HbA1c at baseline (%)	0.059	0.138	0.411	−0.179	−0.418	0.025

*B* partial regression coefficient, *β* standarized partial regression coefficient

In a post hoc power analysis, we had 57 % (between group I and group N) and 51 % (between group D and group N) statistical power to detect a CIMT difference.

## Discussion

Three main findings were obtained in the present study. First, CIMT increased or decreased in diabetic patients undergoing stabilization of glycemic control even in a short period of 2 weeks, and the extent of change could be much larger than the estimated annual increase. Second, baseline FPG and HbA1c levels were highest in group D among the three groups, but the daily glucose profile at baseline and on the day before discharge was not related to the change of CIMT. Third, a decrease of CIMT was associated with a larger baseline CIMT and a decrease of Hb during admission, while an increase of CIMT was associated with a lower baseline HbA1c and minimal change of Hb during admission.

The daily glucose profile, BP, and plasma lipids did not differ among the three groups during admission, suggesting that there was no contribution of these factors to the change of CIMT in the present study. However, baseline FPG and HbA1c were highest in group D, and baseline HbA1c was positively correlated with a decrease of CIMT. Although Hb was not different among the three groups both at baseline and on the day before discharge, baseline Hb was positively correlated with a decrease of CIMT and discharge Hb was negatively correlated with a decrease of CIMT (Table 3). Thus, a higher baseline HbA1c and a greater decrease of Hb during admission were associated with a decrease of CIMT. The decrease of Hb was positively correlated with the baseline HbA1c level according to univariate analysis (r = 0.31, p < 0.001), suggesting that chronic hyperglycemia-induced osmotic diuresis may lead to dehydration and elevation of Hb, which may decrease following alleviation of dehydration due to improvement of hyperglycemia. Thus, in patients with a higher HbA1c accompanied by dehydration, CIMT may decrease after attainment of good glycemic control. On the contrary, baseline Hb was positively correlated with an increase of CIMT, while Hb on the day before discharge and baseline HbA1c were negatively correlated with it (Table 3). Although baseline FPG, HbA1c, and AUCglu were not different between group I and group N, baseline GA (reflecting the glycemic state over 1–2 weeks) was lowest in group I among the three groups. ΔHb was also smallest in group I among the three groups (Fig. 2). These results suggest that mild hyperglycemia baseline and a smaller change of Hb were associated with an increase of CIMT. Although the mechanism of such an increase of CIMT is unclear, it is possible that mild hyperglycemia may induce slight overhydration and a decrease of Hb by hemodilution, which may be associated with a decrease of baseline CIMT, while normalization of hemodynamics by improvement of glycemic control during admission may return CIMT to the level before the onset of recent mild hyperglycemia. It is well known that the Hb level shows fluctuation. In addition to hemodilution, various other factors can influence Hb, such as anemia of chronic disease, alterations of iron stores, chronic blood loss, transfusion, vitamin and mineral status, and seasonal effects [15–19]. However, these factors would affect the Hb over a long period considering that the lifespan of erythrocytes is about 120 days. Thus, the changes of Hb during our study period of <2 weeks may have been due to hemodilution rather than other factors.

Current ultrasound transducers have insufficient axial resolution to separate the intimal and medial layers of the carotid artery wall, which is why the combined intima and media thickness is measured clinically as the CIMT. Pignoli et al. previously compared pathological and in vivo or in vitro B-mode ultrasound measurements using carotid artery autopsy samples from healthy subjects [20]. They revealed that about 20 % of the CIMT is composed of the intimal layer and 80 % is made up of the medial layer, with the intima-media thickness (IMT) values from both in vivo and in vitro B-mode ultrasound measurement being consistent with the results of pathological examination. Some later reports have suggested that the far wall, but not the near wall, of the carotid artery should be assessed in ultrasound studies [21, 22]. While intimal thickening is generally caused by foam cells derived from macrophages or smooth muscle cells infiltrating the subendothelial space and capturing oxidized lipids, medial thickening is induced by smooth muscle hypertrophy and/or hyperplasia [21]. Although the exact mechanism leading to an increase of IMT in diabetic patients remains unclear, Kozakova et al. recently proposed that it may involve chronic hyperglycemia-related enhancement of arterial stiffness and consequent remodeling of the carotid media [11]. They suggested that chronic hyperglycemia induced a decline of arterial wall elasticity and enhanced stiffness, which may then promote adaptive medial thickening by smooth muscle changes, but this mechanism seems unlikely to explain the short-term CIMT changes observed in the present study.

Dehydration due to chronic severe hyperglycemia may lead to an increase of CIMT, while amelioration of dehydration by improvement of hyperglycemia may restore the reversible portion of this increase. On the other hand, mild overhydration due to relatively mild hyperglycemia may decrease CIMT, while amelioration of this state may restore the reversible decrease of CIMT.

Although CIMT is a biomarker of subclinical atherosclerosis, it can fluctuate over a short period due to changes of the blood glucose level or hemodynamics. Therefore, we should consider whether blood glucose and hemodynamics are stable when assessing changes of CIMT.

The present study had several limitations. First, the sample size was small, because the post hoc power analysis showed that this study does not have adequate power which would provide β error. Second, we have no data on healthy subjects. It would probably be difficult to detect short-term changes of CIMT in healthy subjects during daily life because their basal CIMT is smaller than that of patients with diabetes. On the other hand, dehydration induced by exercise would be likely to alter the CIMT, so it may be interesting to investigate whether there are any changes of CIMT after sustained exercise. Third, we did not differentiate between type 1 and type 2 diabetes with respect to the onset, severity of complication, or prognosis. Fourth, we have no data about intra-observer agreement because we only measured CIMT twice over 2 weeks. However, there was no inter-observer variation, because all carotid studies were performed by a single trained examiner and CIMT was automatically determined by using analytical software. Fifth, we did not evaluate biochemical markers of hemodynamic changes, such as brain natriuretic peptide (BNP), plasma renin activity, plasma aldosterone, or fractional excretion of Na, and we did not assess physiological hemodynamic markers like local carotid artery blood flow, wave speed, or stroke volume. Thus, further large-scale studies that investigate such factors will be needed to confirm the above hypothesis.

## Conclusions

CIMT may show plasticity in diabetic patients and can change after even 2 weeks when hyperglycemia is improved. This point should be considered when performing assessment of baseline CIMT and CIMT progression in prospective studies of diabetic patients.

## Abbreviations

CIMT: carotid intima-media thickness
Hb: hemoglobin
CVD: cardiovascular disease
ARIC: atherosclerosis risk in communities
HR: hazard ratio
SD: standard deviation
BW: body weight
BP: blood pressure
FPG: fasting plasma glucose
GA: glycated albumin
SMBG: self-monitoring of blood glucose
CCA: common carotid artery
BMI: body mass index
Ht: hematocrit
AUCglu: area under the curve of daily glucose profile
IMT: intima-media thickness
BNP: brain natriuretic peptide
